# Effects of water absorption on the mechanical properties of hybrid natural fibre/phenol formaldehyde composites

**DOI:** 10.1038/s41598-021-92457-9

**Published:** 2021-06-28

**Authors:** Sekar Sanjeevi, Vigneshwaran Shanmugam, Suresh Kumar, Velmurugan Ganesan, Gabriel Sas, Deepak Joel Johnson, Manojkumar Shanmugam, Athijayamani Ayyanar, Kakur Naresh, Rasoul Esmaeely Neisiany, Oisik Das

**Affiliations:** 1 Department of Mechanical Engineering, Hindusthan Institute of Technology, Coimbatore, Tamilnadu India; 2 Department of Mechanical Engineering, Saveetha School of Engineering, Saveetha Institute of Medical and Technical Sciences, Chennai, Tamilnadu India; 3 Department of Mechanical Engineering, Kalasalingam Academy of Research and Education, Srivilliputtur, Tamilnadu India; 4 Department of Agricultural Engineering, Saveetha School of Engineering, Saveetha Institute of Medical and Technical Sciences, Chennai, Tamilnadu India; 5 Structural and Fire Engineering Division, Department of Civil, Environmental and Natural Resources Engineering, Luleå University of Technology, 97187 Luleå, Sweden; 6 Department of Mechanical Engineering, Government College of Engineering, Bodinayakanur, Tamilnadu India; 7 Department of Mechanical Engineering, Indian Institute of Technology Madras, Chennai, Tamilnadu India; 8 Department of Materials and Polymer Engineering, Faculty of Engineering, Hakim Sabzevari University, 9617976487 Sabzevar, Iran

**Keywords:** Engineering, Materials science

## Abstract

This investigation is carried out to understand the effects of water absorption on the mechanical properties of hybrid phenol formaldehyde (PF) composite fabricated with Areca Fine Fibres (AFFs) and Calotropis Gigantea Fibre (CGF). Hybrid CGF/AFF/PF composites were manufactured using the hand layup technique at varying weight percentages of fibre reinforcement (25, 35 and 45%). Hybrid composite having 35 wt.% showed better mechanical properties (tensile strength ca. 59 MPa, flexural strength ca. 73 MPa and impact strength 1.43 kJ/m^2^) under wet and dry conditions as compared to the other hybrid composites. In general, the inclusion of the fibres enhanced the mechanical properties of neat PF. Increase in the fibre content increased the water absorption, however, after 120 h of immersion, all the composites attained an equilibrium state.

## Introduction

Fibre-based composites have been extensively studied over the past many decades and intensive research into their characteristics and properties has enabled their application in different engineering sectors. However, the use of synthetic fibre composites, such as carbon fibre-based composites and glass fibre-based composites, has detrimental environmental impact due to the problems associated with their production, energy-intensive manufacturing and disposal^1–4^. Natural fibres have been used to overcome these problems and their properties, manifesting in the resulting composites, have been shown to be impressive. In particular, the use of natural fibres over conventional synthetic fibres has been a viable solution due to their low density, good specific strengths and modulus, increased energy recovery, economic viability, reduced tool wear during machining and good biodegradability^5–9^. Natural fibre reinforced composites have been widely used in semi-load bearing, automobile and other outdoor applications, such as exterior underfloor panelling for cars, marine structures and sports equipment^10–13^. As a result, the integration of natural fibres in composites enhanced and Faruk et al.^14^ stated that hemp, flax, jute, ramie, sisal and kenaf fibres were among the most widely used and studied fibre materials from 2000 to 2010. Although natural fibre-based polymer composites have many advantages, there are also certain deficiencies, such as high absorption of moisture, lower strength compared to the synthetic counterparts, incompatibility between fibres and polymer matrices, quality variability, limited processing temperature and lower durability^15–17^. Physical and chemical treatment of fibres is a potential solution to overcome these aforementioned deficiencies. Another possible solution is the application of hybrid composites that can negate some of the disadvantages of ‘single’ natural fibre composites and also facilitate the tailoring of their properties. Appropriate design and selection of fibres with a proper balance between cost and performance could facilitate the development of low-cost high strength hybrid composites^18^. Swolfs et al.^19^ reported that hybridisation can be used to enhance the strength of reinforced fibre composites. Through hybridisation, it is possible to develop composites at a low cost without sacrificing the mechanical and thermal properties^7^. Furthermore, hybrid composites show balanced mechanical strength, which cannot be achieved in single-fibre reinforced composites^2,16^ Atiqah et al.’s^20^ investigation proved that through hybridisation it possible to improve the physical and thermal properties of sugar palm/glass fibre reinforced polyurethane composite. Properties of the hybrid composites are the functions of fibre proportion, fibre orientation, fibre type, fibre properties, fibre to fibre interaction, fibre matrix bonding, fibre size and matrix property^21,22^.

Various studies have been conducted on the preparation of hybrid composites and their performance has been investigated. It has been found that hybrid composites have shown comparable strength to composites with glass fibre reinforcement^16,23–27^. Goud et al.^28^ developed roystonea/glass fibre based epoxy hybrid composites. The hybrid composite showed similar impact strength (168 J/m) as that of the glass fibre epoxy composite (169 J/m). The increased fibre to fibre interaction was the main reason for the improved impact strength in these combinations, and the authors further recommended the roystonea/glass fibre epoxy hybrid composites as a replacement for glass fibre composite in automobile applications. Selver et al.^29^ reported that hybridisation improved the tensile strength of the composites compared to just jute (431%) and flax fibre (292%) reinforced composites. Glass and flax fibre-based hybrid composite showed a maximum tensile strength of 372 MPa. Cavalcanti et al.^30^ fabricated epoxy-based hybrid composites with three different natural fibres, namely, jute, sisal and curaua fibres. Hybridisation enhanced the tensile strength of the composite, wherein, compared to jute fibre composite, hybrid composite having jute and curaua fibre composite showed 77% increased tensile strength and for jute and sisal hybrid composite it was 68%. Hybridisation of sisal and hemp fibre resulted in a 43% increment in the tensile modulus when compared to neat polylactic acid material^31^.

Although natural fibre-based hybrid composites provide enhanced strength, they have poor moisture resistance due to the polar nature of the fibres. This results in the degradation of the fibre–matrix interface region that is severely detrimental to the stress transfer from the matrix to the fibre^32–34^. This is a critical issue in natural fibre hybrid composites that affects the physical, mechanical and thermal properties of the composites^35^. The moisture diffusion in the natural fibre composites is influenced by volume fraction of fibre, humidity, voids, viscosity of matrix and temperature^36^. The poor moisture resistance restricts natural fibre composite application in the aquatic environment and water-based applications^36^. It is therefore mandatory to address and solve this problem in such a way that natural fibre hybrid composites are considered for applications in aquatic environment.

In light of the aforementioned, the present study attempts to investigate the impact of water absorption on hybrid natural fibre composite’s mechanical properties (tensile, flexural and impact). Hybrid composites were manufactured with AFF and CGF in conjunction with a PF matrix. The composites were tested for water absorption characteristics and the water absorbed composites were tested for mechanical properties. The result of the mechanical test was compared to the non-water absorbed composites. This investigation can be useful for the scientific community to comprehend the effect of water absorption on the performance properties of hybrid natural fibre composites and aid in creating designer compositions for use in water-rich applications.

## Materials and methods

### Materials used and composite fabrication

**Figure 1:**
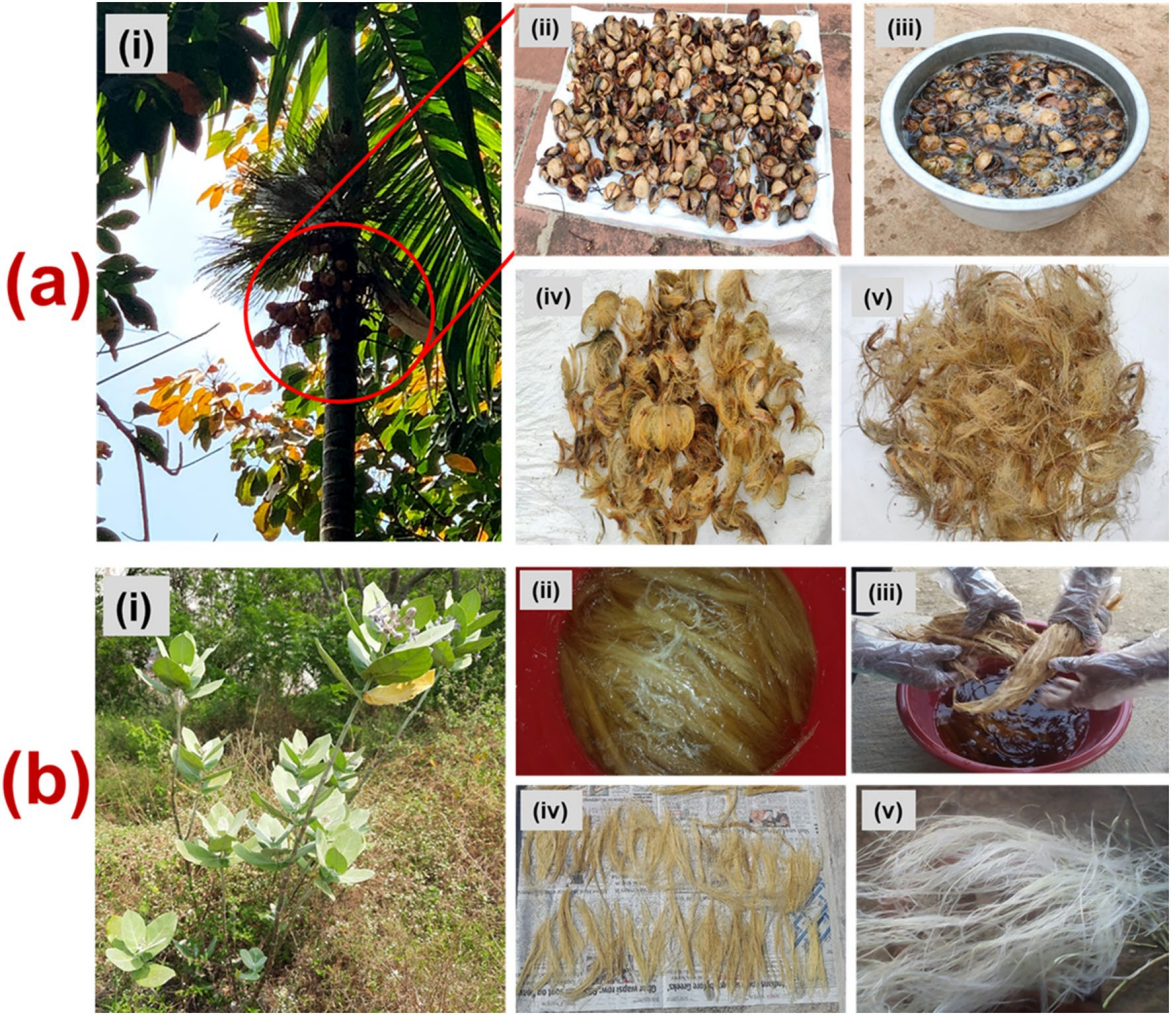
Fibre extraction process (a) Extraction of Areca fine fibre, (i) Areca nut palm plant, (ii) Areca nuts, (iii) retting process, (vi) drying of extracted fibre (viii) fibre after drying. (b) Extraction of Calotropis gigantea fibre, (i) Calotropis gigantea plant, (ii) retting of stem stalks of Calotropis gigantea plant, reproduced with permission form Ref. [3], (iii) cleaning of extracted fibre, reproduced with permission form Ref. [3], (iv) drying of extracted fibre, (v) extracted fibre, reproduced with permission form Ref. [3].

In the present work, two different natural fibres were used, namely, *Calotropis gigantea* fibre and *Areca* fine fibre. *Calotropis gigantea* fibre was extracted from the stem of the plant, and *areca* fibre was extracted from the matured areca nut husk. Both the fibres were extracted manually via retting process. Figure 1 depicts the fibre extraction process for the *areca* fine fibre and *Calotropis gigantea* fibre. Both fibres used were short fibres having a length of 9 ± 1 mm. For composite fabrication, phenol-formaldehyde (PF) resin (CAS number 9003-35-4) was used along with the cross-linking agent (divinylbenzene) and acidic catalyst (hydrochloric acid), purchased from M/s POOJA Chemicals, Madurai, India. The PF used has a specific gravity of 1.7 and a processing temperature range of 149–153 °C.

Hybrid composites were manufactured using a hand layup technique. Before manufacturing, the fibres were dried by placing them in an oven at 60 °C for 24 h to ensure a bone-dry nature. The required quantity of matrix PF with a cross-linking agent (divinylbenzene) and an acidic catalyst (hydrochloric acid) were mixed at a ratio of 100:2:1.5 and was poured over the pre-weighted fibres placed in the mould cavity. Fibres were included in 1:1 ratio in order to have the same contribution level of the two fibres. A steel mould of size 200 × 150 × 3 mm was used, which was coated with wax for easy removal of composites. After applying the matrix, the mould was left free for a few minutes to ensure the infiltration of the matrix into the fibres. By using a roller, the trapped air was removed. Immediately after that, the mould was closed and a pressure of 100 kg/cm^2^ was applied. The mould was eventually left undisturbed and allowed to cure at room temperature for 24 h. Details of the hybrid composite fabrication are shown in Table 1.

**Table 1:** Details of fabricated composite.

S. no.	Composite fabricated	Fibre reinforcement (%)		Matrix reinforcement (%)
		CCF	AFF	
1	Hybrid composite with 25 wt.% fibres	12.5	12.5	75
2	Hybrid composite with 35 wt.% fibres	17.5	17.5	65
3	Hybrid composite with 45 wt.% fibres	22.5	22.5	55

### Characterisations

#### Mechanical tests

The tensile test was carried out using the universal testing machine (Make—Fuel Instruments and Engineers Pvt. Ltd, Model—UTE 120) at a cross-head speed of 2 mm/min and a gauge length of 50 mm, according to ASTM D 638-10^37^ protocol. Flexural testing was carried out according to ASTM D 790-10^38^ standard using a three-point bending rig at a crosshead speed of 2 mm/min and a gauge length of 50 mm. The impact test was performed in accordance with ISO 180^39^ protocol using an Izod impact machine. All the tests were performed at room temperature. Five samples were tested for each combination and the average values were reported. To find the effect of water absorption on the mechanical properties of CGF/AFF/PF hybrid composites, the tensile, flexural and impact test was conducted with the same procedure. The water absorbed samples were dried and then used for experiments.

#### Water absorption test

The water absorption test for CGF/AFF/PF hybrid composites was carried out in accordance with ASTM 570^40^ protocol by soaking in distilled water at room temperature for 10 days. Prior to the experimentation, hybrid composites were placed in an oven at 60 °C for 24 h, to ensure no residual moisture remained and weighed using a precision balance. Immediately after the weighing of the composites, they were immersed in water. After every 24 h, the composite specimens were periodically taken from the water bath and the specimen surface was wiped using tissue paper. After that, the composite specimens were weighed to determine the mass of the water they absorbed. Composite specimens were weighed regularly until the water absorption percentage attained a saturation level. In the present investigation, all the composites attained saturation at 10 days of immersion. After the 10th day there was no increase in the water absorption percentage, hence, for analysis, water absorption percentage for 10 days is reported (24, 48, 72, 96, 120, 144, 168, 192, 216 and 240 h). The percentage of water absorption is measured using the following formula (Eq. 1)^36^:

1 $$WA\;(\% ) = \frac{{W_{1} - W_{0} }}{{W_{0} }} \times 100$$

where WA is the water absorption, w_1_ is the weight of the composite after water immersion and w_0_ is the weight of the composite before water immersion.

Using various modelling methods, the water absorption of the composites can be predicted by means of which the diffusivity of the water molecules can be found in the composites. Such modelling methods use empirical and analytical formulations and Fickian diffusion is a popular analytical method for finding the water absorption of fibre composites. According to the Fickian diffusion law, the moisture content is expressed in terms of time, diffusion coefficient, moisture percentage in the saturation period and thickness of the material, which can be expressed as in Eq. (2)^41–43^:

2 $$C(t) = C_{s} - {\frac{8C_{s}}{\pi ^{2}}} \mathop \sum \limits_{{K = 0}}^{{\infty}} \frac{1}{{(2K + 1)^{2} }}\exp \left( { - \frac{{(2k + 1)^{2} D\pi ^{2} }}{{d^{2} }}t} \right)$$

where *c*(*t*) is the total moisture content, *D* is the diffusion coefficient, *C*_*s*_ is the moisture content in the saturation time, *k* is the constant, *t* is the specific time and *d* is the thickness of the material. The water absorption characteristics of the CGF/AFF/PF hybrid composites can be studied using the following parameters: diffusion coefficient, sorption coefficient, and permeability coefficient. The amount of water molecules absorbed by the composites depends on the diffusion of water molecules. The diffusion of water molecules can be studied through the parameter diffusion coefficient (D), which can be numerically calculated using Fick’s diffusion coefficient as per Eq. (3). Sorption coefficient (Sc) in Fickian diffusion law defines the rate at which the water molecules are absorbed by the composites, which can be calculated using Eq. (4). Permeability coefficient (Pc) is another important parameter in Fickian diffusion law, which quantitatively measures the rate at which a water molecule penetrates a composite. Using Eq. (5), the permeability coefficient of a composite can be calculated^44^.

3 $${\text{Diffusion}}\;{\text{coefficient}}\;({\text{D}}) = \pi \left( {\frac{{{\text{mh}}}}{{4{\text{W}}_{\infty } }}} \right)^{2}$$

where ‘*h*’ is the thickness of hybrid composite specimens and ‘*m*’ is the slope of the linear portion of the sorption curve Sorption coefficient (*Sc*).

4 $${\text{Sorption}}\;{\text{coefficient}}\;({\text{Sc}}) = \frac{{{\text{W}}_{\infty } }}{{{\text{W}}_{{\text{t}}} }}$$

where *W*_*∞*_ and *W*_*t*_ are the percentages of water absorption at saturation time and at a specific time *t , respectively*.

5 $${\text{Permeability}}\;{\text{coefficient}}\;(Pc) = Sc \times D$$

where *Sc* is the sorption coefficient, and *D* is the diffusion coefficient.

#### Electron microscopy

The microstructure of the CGF/AFF/PF hybrid composites was analysed using a scanning electron microscope (SEM) Hitachi-S3000N. SEM images were taken at the voltage of 20 kV and the working distance was varied between 30 to 40 mm. Samples were prepared from the fractured (tensile, flexural and impact) composite surface to analyse the fracture morphology. The sample of 5 mm in height was cut from the fractured end of the composite and was used for imaging.

## Results and discussions

### Water absorption behaviour

Lignocellulosic fibres have poor resistance to moisture absorption and, due to this natural fibre reinforced composites have undesirable effects on dimensional stability and mechanical properties when exposed to moisture in the environment. In order to understand the durability of composites based on the field of application, it is necessary to study the moisture absorption behaviour of natural fibre composites. In general, water molecules penetrate natural fibre composites by three different mechanisms: diffusion of moisture content within the micro-gaps between the polymer chains; capillary transport into the micro-gaps; and flaws in the interfaces between the fibres and the matrix^45–47^. The moisture is mainly absorbed by the interface between the fibre and the matrix, as well as by the fibre itself via hydrogen bonding.

**Figure 2:**
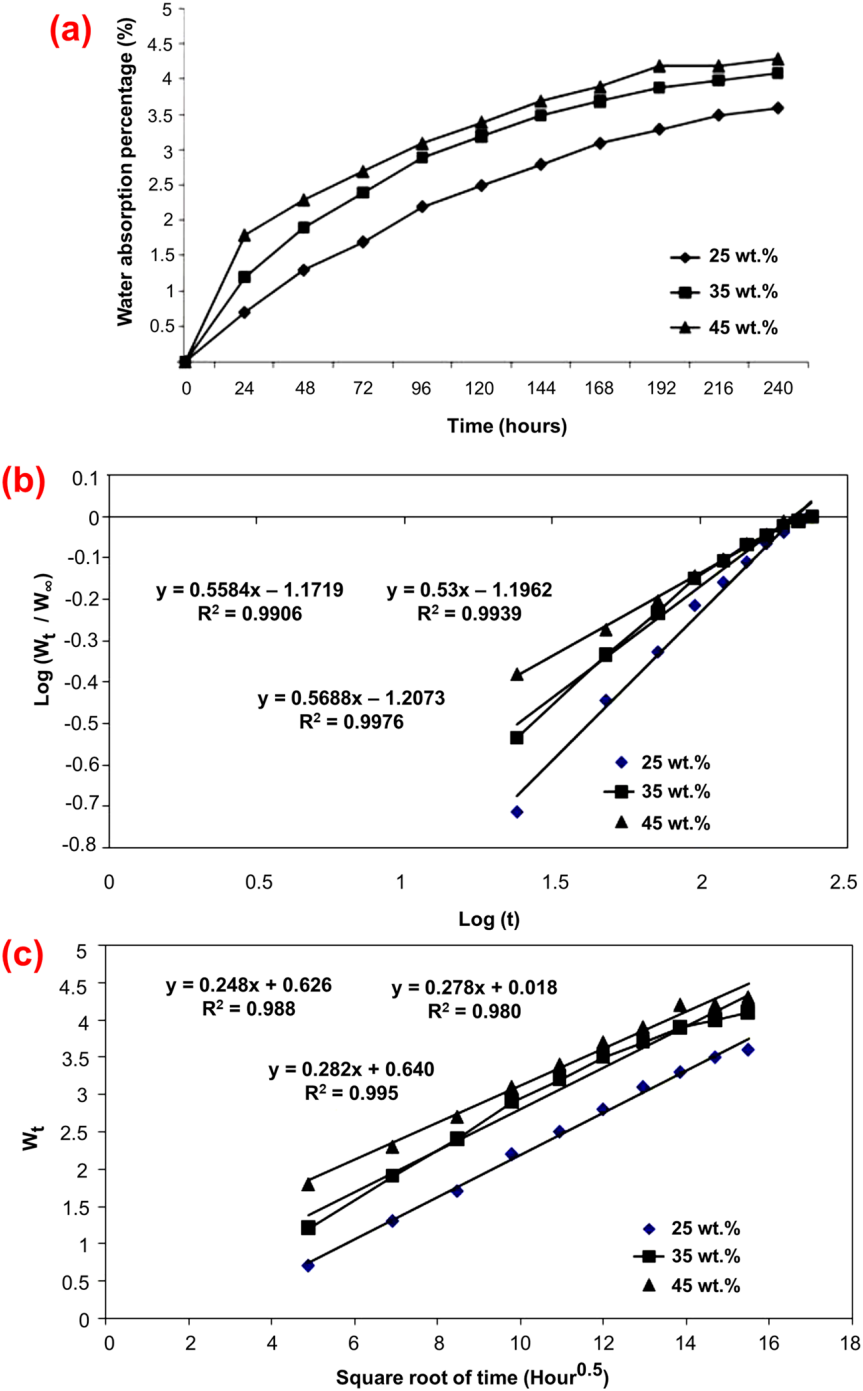
(**a**) The water absorption percentage versus (vs.) immersion time curves of CGF/AFF/PF hybrid composites, (**b**) diffusion curve fitting plots for CGF/AFF/PF hybrid composites, and (**c**) diffusion curve fitting plot between *Wt* and square root of time for CGF/AFF/PF hybrid composites.

The percentage of water absorption was plotted against the square root of time (hours) as shown in Fig. 2a. The results were expected because the higher the natural fibre content in the composite, the higher the water absorption rate. The penetration of water molecules into the composite specimens was calculated using Eq. (1). The percentage of water absorption increased with immersion time and became stable after 10 days. From Fig. 2a, it is observed that the hybrid composite with 45 wt.% fibre reinforcement showed the maximum water absorption percentage of 4.3%, followed by the hybrid composite with 35 wt.% (4.1%). This difference between the 35 wt.% and 45 wt.% hybrid composites is due to the weight percentage of the fibres. It can be also seen that the amount of water absorbed by 25 wt.% of the hybrid composite was less than that of 35 and 45 wt.% of hybrid composites. Higher reinforcement increased water absorption due to increased contact between water molecules and fibre. In addition, the percentage of water absorption increased linearly from the beginning of the immersion period, indicating the steady absorption of water by natural fibre composites. Similar results were observed in napier grass fibre/polyester composites^48^. However, in that study, long and short napier grass fibre were used and reinforced at a volume percentage of 25%. The long fibre composite exhibited the highest water absorption of ca. 20%. Based on the findings of the current study, it can be speculated that hybridisation in the napier grass fiber/polyester composite will result in a reduction in water absorption.

For all the hybrid composites exposed to distilled water, the amount of water penetration increased linearly and reached a saturation state after 10 days of immersion. From the water absorption results, it is clear that the lowest and the highest percentage of water absorption was observed at 25 wt.% of hybrid composite and 45 wt.% of hybrid composite, respectively. Compared to 35 wt.% and 45 wt.% of the composites, 25 wt.% of the composites showed reduced water absorption, and this is because the amount of matrix is higher at the lower fibre weight percentage. The matrix material is hydrophobic, which restricts the interaction of the water molecules with the fibre content. Aji et al.^49^ found that after 14 days of immersion, kenaf/pineapple leaf fibre composites had a minimum water absorption of 4%. This was accomplished by varying the kenaf as well as the pineapple leaf fibre reinforcement. In the current study, both fibres were reinforced in an equal proportion; however, by optimising the individual fibre reinforcement, it is possible to reduce water absorption even further.

Water molecules penetrate into the hybrid composite by the diffusion mechanism, and the diffusion coefficients with the sorption and permeability coefficients can be calculated using the experimental data. It has been reported that the diffusion coefficient can be used to determine the permeability of water molecules. For example, in the study of Vigneshwaran et al.^36^, sisal fibre composites reached equilibrium quickly when the diffusion rate was at its maximum.

Figure 2b shows the diffusion curve fitting plot, log (W_t_/W_∞_) vs. log (t), for CGF/AFF/PF hybrid composites exposed to distilled water. Table 2 gives the values of the linear (*k*) and angular (*n*) coefficients obtained from the plot for CGF/AFF/PF hybrid composite. The value of the linear coefficient ‘*k*’ can be obtained from Fig. 2b and Table 2. The similarity between the hybrid composite materials and water contents can be identified by the linear coefficient ‘*k*’. The higher value of ‘*k*’ represents strong similarity. From Table 2, it can be seen that the ‘*k*’ values exposed to the distilled water environment are close to 1.2 for all the CGF/AFF/PF hybrid composites. The values of the coefficient of determination ‘*R*^2^’ represent the relationship and found to be in good agreement. Similar results were obtained in the investigation of the akund fibre’s (*Calotropis*—cellulose fibre) water absorption behaviour, where the ‘R^2^’ value was 0.99^50^.

**Table 2:** The obtained values of constants in water absorption for all hybrid composites.

Hybrid composites fibre content (wt.%)	Constants		
	n	k	R^2^
25	0.5584	1.1719	0.9906
35	0.5300	1.1962	0.9939
45	0.5688	1.2073	0.9976

Figure 2c illustrates the diffusion curve fitting plot between *W*_*t*_ and square root of time for CGF/AFF/PF hybrid composites. Table 3 gives the maximum level of water absorption and calculated values of the coefficient of diffusion ‘*D*’ for the hybrid composites. The almost same water diffusion was identified for all the hybrid composites, as shown in Fig. 2c and Table 3. The diffusion coefficients increased with the increase of CGF and AFF fibre content. From the observation, it can be inferred that the penetration of water molecules into the composite is through *non-Fickian* diffusion process. The diffusion, sorption, and permeability coefficient of CGF/AFF/PF hybrid composites are presented in Table 4. It can be seen that 25 wt.% of hybrid composite shows the lowest percentage of water absorption and swelling thickness as compared to the other tested hybrid composites due to the fibre contents. It is also observed that the hybrid composite with 25 wt.% of fibre contents show the lowest coefficient values. Similar findings were observed in the study of Vigneshwaran et al.^36^, where it was reported that at higher fibre weight percentages, composites readily absorbed water molecules, resulting in increased water absorption and swelling.

**Table 3:** Maximum percentage of water absorption and diffusion coefficients for all CGF/AFF/PF hybrid composites.

Hybrid composite fibre content (wt.%)	Maximum % of water absorption	m	k	R^2^	D (× 10^–2^ m^2^ s^−1^)
25	3.6	0.248	0.626	0.988	0.0941
35	4.1	3.47	0.018	0.980	0.1126
45	4.3	3.72	0.995	0.995	0.1147

**Table 4:** Diffusion, sorption, and permeability coefficients for all CGF/AFF/PF hybrid composites.

Hybrid composite fibre content (wt.%)	Absorption coefficient	Diffusion coefficient D (× 10^–2^ m^2^ s^−1^)	Permeability coefficient P (× 10^–2^ m^2^ s^−1^)
25	2.95	0.0941	0.2776
35	3.47	0.1126	0.3907
45	3.72	0.1147	0.4267

### Effect of water absorption on tensile strength

**Figure 3:**
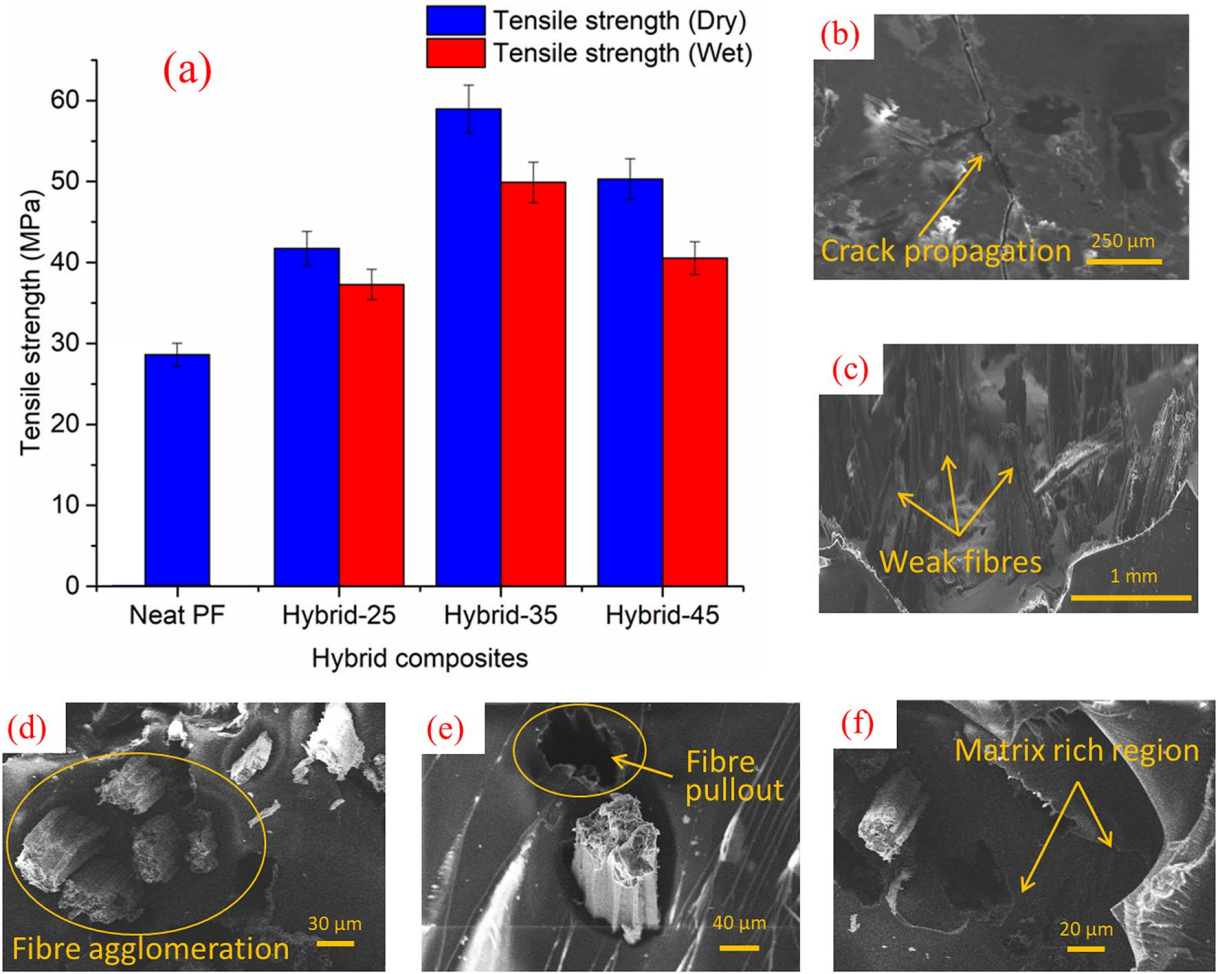
(**a**) Effects of water absorption on the tensile strength of CGF/AFF/PF hybrid composites based on the weight percentages of fibre. (**b**,**c**) SEM image of the fractured surface of the 35 wt.% hybrid composite after the tensile test (wet condition), (**d**,**e**) SEM image of the fractured surface of the 45 wt.% hybrid composite after the tensile test (wet condition), (**f**) SEM image of the fractured surface of the 25 wt.% hybrid composite after the tensile test (wet condition).

The tensile properties vs. fibre weight percentage results for CGF/AFF/PF hybrid composite specimens are shown in Fig. 3a at without (dry) and with water absorption (wet) conditions. The tensile properties of the hybrid composites decreased drastically on exposure to the distilled water immersion. Compared to 25 wt.%, the tensile strength was found to increase significantly as the fibre weight percentage increased to 35 wt.% after that decrement was noted at 45 wt.% reinforcement. The reduction in the strength at 45 wt.% composite was due to the agglomeration of fibres. A similar trend was also observed in the hybrid composites at the wet condition. The maximum tensile strength of hybrid composites at wet condition was ca. 50 MPa. The tensile strength of 35 wt.% composite at wet condition was ca. 18% lower than the 35 wt.% composite at dry condition. When compared with the dry hybrid composites, the composites at wet condition show a lower range of mechanical properties. It was due to the water immersion of composite specimens in which the hydrogen bonding is formed between the water molecules and cellulose. The penetration of water molecules into the fibre–matrix region led to the change of dimensions of the composite specimens and caused weak interfacial bonding, thereby decreasing the tensile properties. Dimensional variation occurred due to the swelling of the fibre, which led to fibre detachment from the matrix, creating weak fibre matrix bonding. The weak interface reduced the composite elongation property leading to failure due to crack propagation, as shown in Fig. 3b. Figure 3c shows fibre fracture that was induced by water absorption, which made them weak. Figure 3d,e show the fibre pullout and fibre agglomeration in the 45 wt.% composite (wet condition). Higher amount of fibre reinforcement lead to agglomeration, which resulted in poor load distribution and failure. Matrix rich area in the 25 wt.% composite is highlighted in the Fig. 3f. Due to reduced fibre content there was a poor load transfer, which led to brittle failure in the matrix rich surface. According to the findings of Saidane et al.^26^, the swelling of natural fibres in composites due to water absorption causes shear stresses in the matrix interface, resulting in delamination, which could also explain the lower strength of water-absorbed composites.

### Effect of water absorption on flexural strength

**Figure 4:**
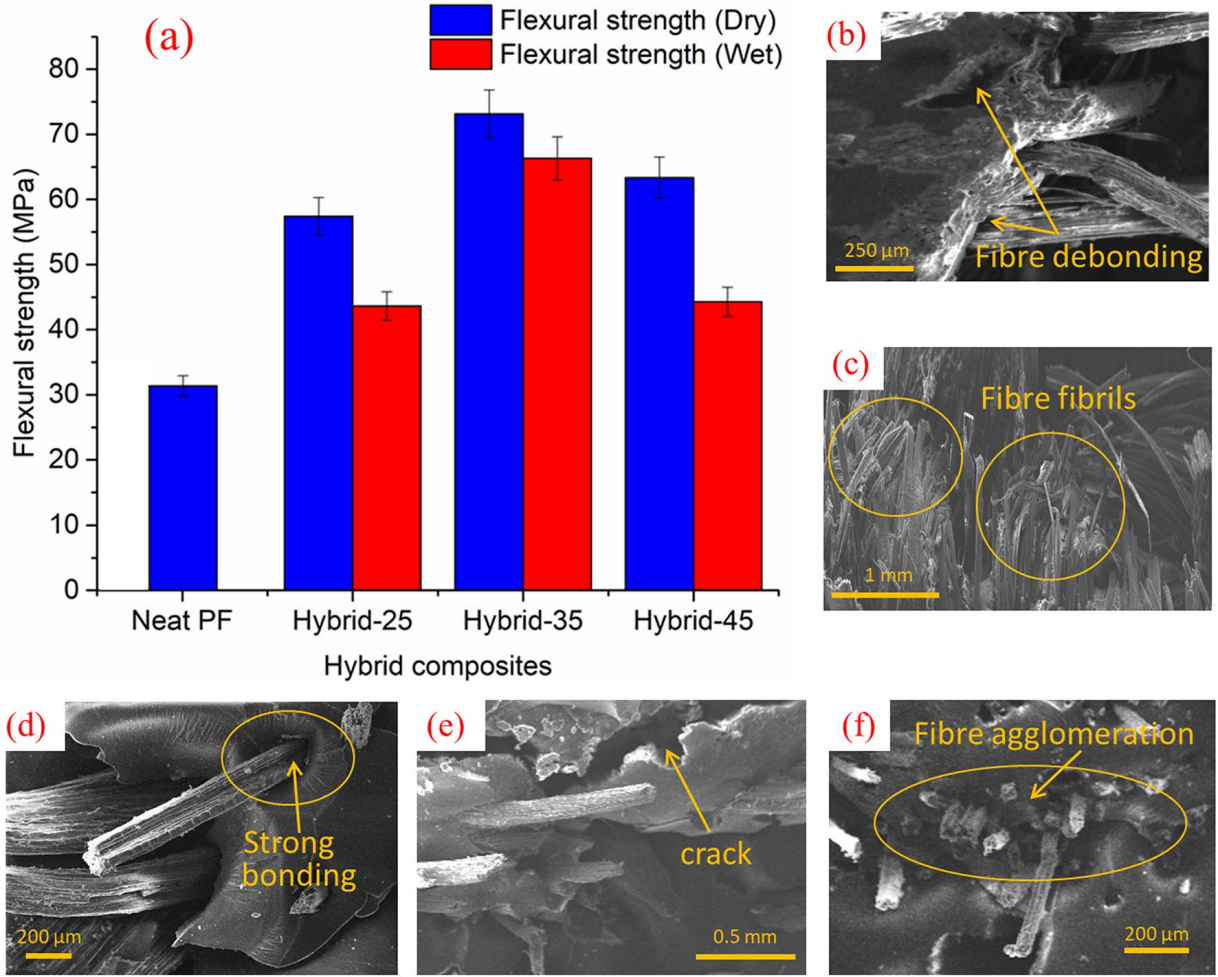
(**a**) Effects of water absorption on the flexural strength of CGF/AFF/PF hybrid composites based on the weight percentages of fibre. (**b**,**c**) SEM image of the fractured surface of the 35 wt.% hybrid composite after the flexural test (wet condition), (**d**) SEM image of the fractured surface of the 35 wt.% hybrid composite after the flexural test (dry condition), (**e**,**f**) SEM image of the fractured surface of the 45 wt.% hybrid composite after the flexural test (wet condition).

Figure 4a shows the results of the flexural strength of hybrid composites at dry and wet conditions. The hybrid composites showed a similar trend as tensile strength at the dry and wet condition for the flexural strength. For the dry samples, the flexural strength increased with the increase of hybrid fibre content up to 35 wt.% and then dropped at 45 wt.% of hybrid fibre. The 35 wt.% composites showed enhanced bonding between fibre and matrix compared to other composites (Fig. 4d). During a 3-point flexural test, one side (convex) of the composite experiences tension while the other side (concave) experiences compression. This resulted in lower tensile strength in 45 wt.% fibre added composites, which also caused lower flexural strength. Furthermore, increased fibre content reduces fibre wetting. At wet condition, the maximum flexural strength value was also obtained at 35 wt.% (similar to tensile strength), which is 10% lower than the dry composite at 35 wt.%. It can be seen that the composite specimens at wet condition have lower flexural strength values compared to flexural strength values of the composite specimens at the dry condition. It was due to the swelling of the fibres as a result of the penetration of water molecules in the interfacial region between the fibre and the matrix. Due to this, gaps formed between the fibre and the matrix (Fig. 4b), which led to the de-bonding of the fibre from the matrix. The presence of moisture in the fibre degrades them, separating the fibres into fibrils, which can be observed in Fig. 4c. Hence, the flexural strength of the hybrid composites at wet condition was lower. Furthermore, at 45 wt.%, fibre agglomeration (Fig. 4f) was also noted that caused detrimental effect on the flexural strength. Maslinda et al.^46^ found that the variation in the fibre matrix interface was primarily responsible for the reduction in the flexural properties of water absorbed composites. It was also reported that when water molecules penetrated into the macro-voids and free space of the matrix, new cavities and cracks formed (Fig. 4e), acting as a water transport pathway within the composites and reducing interfacial bonding.

### Effect of water absorption on impact strength

**Figure 5:**
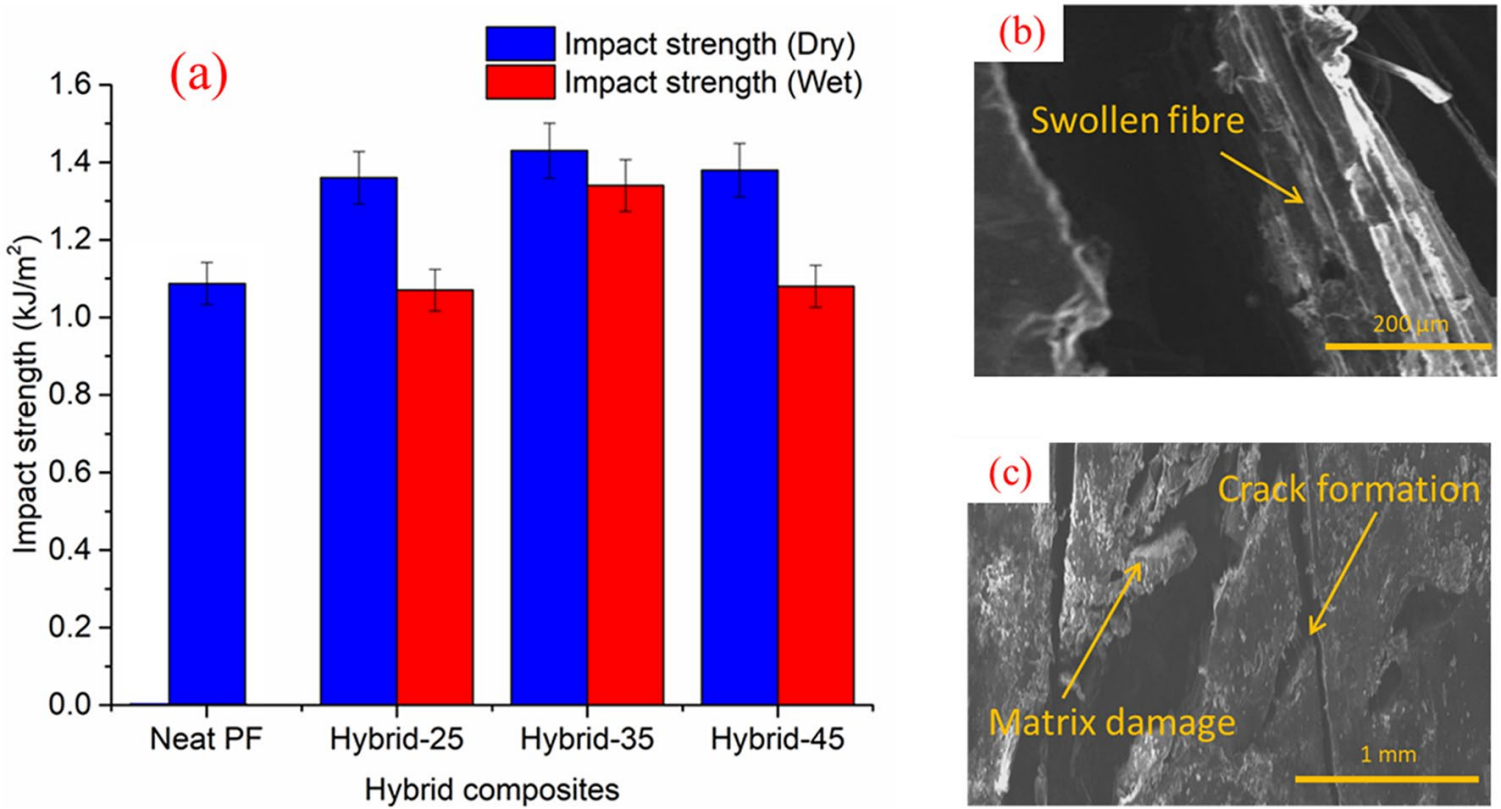
(**a**) Effects of water absorption on the impact strength of CGF/AFF/PF hybrid composites based on the weight percentages of fibre. (**b**,**c**) SEM image of fractured surface of the 35 wt.% hybrid composite after the impact test (wet condition).

The results of the impact strength of hybrid composite at dry and wet conditions are shown in Fig. 5a. For the dry composite specimens, the impact strength increased as hybrid fibre content increased up to 35 wt.% and then decreased. At wet condition, the impact strength of composite decreased after 35 wt.% of hybrid fibres. When compared with the impact strength of dry specimens at 35 wt.%, composite specimen at 35 wt.% of hybrid fibres shows 7% of reduction. It is clear that the impact strength of hybrid composites at wet condition is lower than the impact strength of hybrid composites at dry condition. This is due to the penetration of water molecules into the interface region, leading to the formation of a higher number of microcracks as a result of fibres’ swelling, as shown in Fig. 5b,c. This result was similar to the findings of Maslinda et al.^46^ and Saidane et al.^26^. A similar failure mechanism was observed in both the investigations, and it was reported that the failure was primarily caused by poor bonding between the fibre and the matrix.

## Conclusions

The CGF/AFF/PF hybrid composites were prepared by varying fibre concentrations using the hand lay-up technique. The effects of water absorption on the mechanical properties (tensile, flexural and impact) of CGF/AFF/PF hybrid composites have been studied. The percentage of water absorption increases with the fibre weight percentage increase. The water absorption behaviour of this composite follows the *non-Fickan*’*s* law. The mechanical properties of the hybrid composites at wet condition had lower values than the composites at dry condition. In both the wet and dry condition, hybrid composite with 35 wt.% fibre reinforcement showed maximum strength. Composite with 35 wt.% fibre reinforcement exhibited better fibre–matrix bonding than the other two composites. Formation of agglomeration at 45 wt.% composite reduced their strength compared to 35 wt.% composites. The water penetration inside the fibre compromised the interfacial bonding leading to poor strength for those composites. On the other hand, 25 wt.% was inadequate to cause a significant reinforcement effect.
